# Stem cell transplantation improves aging-related diseases

**DOI:** 10.3389/fcell.2014.00016

**Published:** 2014-05-02

**Authors:** Susumu Ikehara, Ming Li

**Affiliations:** Department of Stem Cell Disorders, Kansai Medical UniversityHirakata, Osaka, Japan

**Keywords:** bone marrow transplantation, type 2 diabetes mellitus, osteoporosis, Alzheimer's disease, aging-related diseases

## Abstract

Aging is a complex process of damage accumulation, and has been viewed as experimentally and medically intractable. The number of patients with age-associated diseases such as type 2 diabetes mellitus (T2DM), osteoporosis, Alzheimer's disease (AD), Parkinson's disease, atherosclerosis, and cancer has increased recently. Aging-related diseases are related to a deficiency of the immune system, which results from an aged thymus and bone marrow cells. Intra bone marrow-bone marrow transplantation (IBM-BMT) is a useful method to treat intractable diseases. This review summarizes findings that IBM-BMT can improve and treat aging-related diseases, including T2DM, osteoporosis and AD, in animal models.

## Introduction

Aging is a complex process of damage accumulation, and has been viewed as experimentally and medically intractable. The process of aging leads to marked malfunction of multiple cellular and molecular events that ultimately get translated into various chronic ailments and diseases such as Type 2 diabetes mellitus (T2DM), Alzheimer's disease (AD), and osteoporosis, Parkinson's disease, atherosclerosis and cancer (Caruso et al., 2004). In this review, we summarize the findings in animal model mice treated with stem cell transplantation for T2DM, osteoporosis and AD. T2DM is induced by obesity, a sedentary lifestyle and nutritional factors, while there are also genetic factors that appear to impact the interaction of multiple genes during the development of T2DM (Adeghate et al., 2006; Ali, 2013). T2DM is not considered to be an autoimmune disease, but some autoantibodies such as islet-cell antibodies and glutamic acid decarboxylase antibodies have been reported to be positive in some young T2DM patients (Klingensmith et al., 2010). Therapies for T2DM mainly include lifestyle changes and oral drugs to reduce the hyperglycemia and improve insulin sensitivity. However, these measures fail to maintain blood glucose levels in the normal range all the time. Recent research into cell based-stem cell therapies has been focused on T2DM. Osteoporosis is one of the most common bone disorders and is now classified into primary and secondary types. Though primary osteoporosis usually occurs in both sexes at all ages, it is often observed in postmenopausal women and even in men later in life. A review of current therapies indicates that bisphosphonates, anti-receptor activator of NF-kb ligand, and anti-sclerostin antibodies are used to counter osteoporosis (Das and Crockett, 2013). AD is a kind of neurological disorder that causes a decrease in cognitive ability, resulting from the deposition of beta-amyloid plaque, neurofibrillary tangles and neurodegeneration (Mielke et al., 2014). The role of tau has become the focus of attention in AD therapy, because current drug treatment using cholinesterase inhibitors or NMDA antagonists has proven to be only modestly successful (Medina and Avila). Animal models are useful for basic studies, and the senescence-accelerated mouse (SAM) strain was established as a novel murine model of senescence acceleration and age-associated disorders (Takeda, 1999). This strain includes SAM-prone, short-lived mice (SAMP) and SAM-resistant, long-lived mice (SAMR). The respective SAMP models, with their characteristic pathological phenotypes, show similar age-associated disorders, including osteoporosis and AD, to those often observed in elderly humans (Takeda, 2009).

## Aging impairs immune response and thymus

It is widely accepted that progression of age is associated with an increasingly compromised immune system. Age-related hematologic changes are reflected by a decline in bone marrow cellularity and a declining adaptive immunity (Linton and Dorshkind, 2004; Hakim and Gress, 2007). Aging is associated with profound alterations in the innate immune system, as exemplified by alterations in the T cell and B cell compartments, functional decline in the monocytes and macrophages, low expression of Toll-like receptors from activated splenic and peritoneal macrophages, and an altered secretion of several chemokines and cytokines (Licastro et al., 2005). Additionally, aged dendritic cells have been reportedly found to be less efficient in activating T and B cell populations (Meydani and Wu, 2007). Key manifestations of changes in the immune system that progress with age are reduced efficacy of vaccine-induced protection against infections/diseases and poor response to new pathogens (Nikolich-Zugich, 2008). Th17+ CD4 cells increased in the aged mice resulting from elevation of IL-1β expression and the reduction in IL-2 expression in aged mice (Lim et al., 2014). Restoration of the T-cell population balance and numbers has been shown to lead to a marked improvement in immunogenic response (Haynes et al., 2005). Alterations in B cells have also been recognized in age-related changes in the immune system. In elderly humans, peripheral B cell percentages and numbers are significantly lowered and show decreased humoral immunity to pathogens and vaccines (Frasca et al., 2008).

The thymus undergoes age-related progressive involution with decreased thymic lymphopoiesis, reduced thymic size and disrupted thymic architecture (Li et al., 2003). The thymus is mainly composed of T-cell precursors-thymocytes of hematopoietic origin and thymic stroma cells (TSCs) of non-hematopoietic origin, which are primarily thymic epithelial cells (TECs). The thymus, which is the source of mature T lymphocytes, involutes steadily with increasing age, which results in a decreased release of new naïve T cells to the periphery, thereby affecting the adaptive immunity (Aspinall, 1997). T cell differentiation and repertoire selection are mediated by specialized cellular microenvironments provided by the TSC. There are reduced numbers of all thymocyte subpopulations from early T-cell progenitors to double-negative, double-positive, CD4 and CD8 single-positive populations with aging (Heng et al., 2005). Our previous report showed the existence of donor-type stromal cells in the thymus of mice was when treated with allogenic bone marrow transplantation (BMT) plus bones. These findings strongly suggest that stromal cells can migrate from the bone marrow (BM) to the thymus, where they participate in the positive selection of thymocytes (Li et al., 2000).

**Figure 1:**
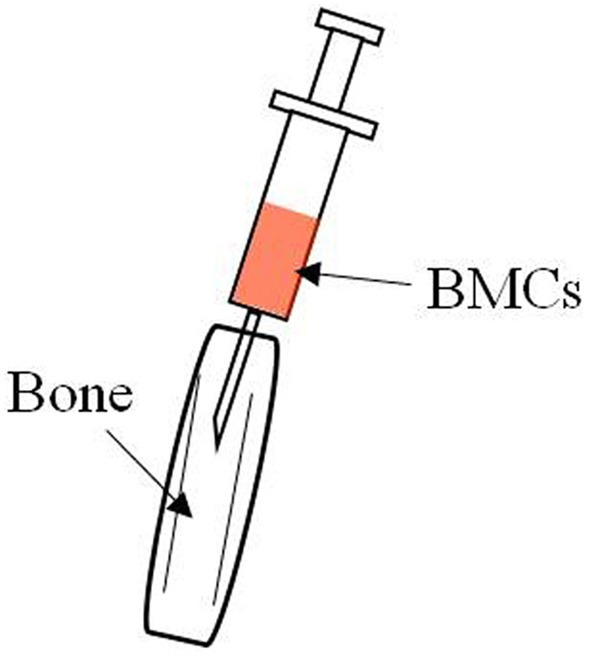
Hair on the knee joint was shaved and a 26-gauge needle was inserted into the bone marrow cavity to make a hole in the bone. A microsyringe containing the donor BMCs was then inserted and the BMCs injected via the bone hole into the bone marrow cavity.

Donor- derived TECs were found in both the medullary and the cortical areas of the thymus in MRL/lpr mice treated with allogenic intra bone marrow-BMT (IBM-BMT, Figure 1). Furthermore, BM cells contain the precursors of functional TECs, and they can differentiate into TECs, which results in the correction of thymic function (Takaki et al., 2008).

## Stem cell therapies for T2DM

T2DM shows not only a deficiency in insulin sensitivity but also reduced beta cell mass, the beta cell mass in T2DM patients being only 40–60% of the normal level (Guo et al., 2013). Stem cell therapies for T2DM will likely be a useful approach in the clinical environment. Hyperglycemia was reversed in streptozotocin-treated diabetic mice when embryonic stem cell (ESC)-derived insulin-producing cells were transplanted into these mice (Raikwar and Zavazava, 2012). Human ESCs effectively differentiated into islet-like cells when exendin-4 was added, and these insulin producing cells ameliorated hyperglycemia in NOD/SCID diabetic mice when transplanted into these mice (Bose et al., 2012). However, there are ethical issues when using human embryos, and ESC transplants into mouse models can result in malignant tumors.

Yamanaka's group was the first to report in 2006 the inducement of pluripotent stem cells (iPSCs) from mouse embryonic or adult fibroblasts (Takahashi and Yamanaka, 2006). Human iPSCs were derived from skin cells by retroviral expression of OCT4, SOX2, c-MYC, and KLF4, and have since been shown to differentiate into functional insulin-producing cells (Tateishi et al., 2008). iPSCs can differentiate into insulin-producing cells responding to glucose stimulation, thereby improving the hyperglycemia in T1DM and T2DM mouse models (Alipio et al., 2010). For preventing allograft rejection in the clinical setting, iPSCs have been generated from diabetes patients themselves, including those with T1DM and T2DM as well as those with other types of diabetes such as mature-onset diabetes of the young (Maehr et al., 2009; Ohmine et al., 2012; Teo et al., 2013).

Autologous BM-derived rat MSCs were expanded *in vitro*, and transplanted into rats in which the diabetic state was induced by streptozocin (STZ). Transplanted MSCs can home to the pancreas and promote PDX-1 and insulin expression in the islets to normalize hyperglycemia, and these MSCs have immunoregulatory effects (Boumaza et al., 2009). Human BM-derived MSCs have been shown to protect human islets from pro-inflammatory cytokines (Yeung et al., 2012), and to enable MSCs to differentiate into insulin-producing cells *in vitro*. These cells have been shown to improve hyperglycemia when transplanted into diabetic mice (Gabr et al., 2013). Multiple intravenous bone marrow-derived MSC injections normalized hyperglycemia in rats in which T2DM was induced by a high fat diet and STZ (Hao et al., 2013). Human adipose tissue-derived MSCs also differentiate into glucose-sensitive insulin-producing cells, which help improve glucose levels and decrease levels of inflammatory cytokines and free fatty acids in T2DM mice (Dave et al., 2013; Nam et al., 2013). Umbilical cord blood-derived-MSCs have also been shown to differentiate into insulin-producing cells, and these cells expressed pancreatic beta cell development-related genes. Moreover, these differentiated insulin-producing cells were able to alleviate hyperglycemia after being transplanted into diabetic NOD mice (Wang et al., 2011a). Clinical data show that umbilical cord blood-derived stem cells reverse the immune dysfunction via the modulation of the immune response in T2DM patients (Zhao et al., 2013).

We previously described how BM-derived stem cells can ameliorate blood glucose levels in KK-Ay mice, a T2DM mouse model (Li and Ikehara, 2013). We also showed that IBM-BMT combined with Co (III) Protoporphyrin IX Chloride, which induces HO-1 expression, could eradicate T2DM in an ob/ob mouse model (Abraham et al., 2008). We used not only IBM-BMT but also thymus transplantation (TT) to treat the db/db mouse, another T2DM mouse model, because the db/db mouse exhibits a marked reduction in the size and cellularity of the thymus (Kimura et al., 1998). Our results showed that, in this mouse model, IBM-BMT+TT increased insulin sensitivity and decreased blood glucose levels resulting from a normalization of the ratio of CD4/CD8 in the peripheral blood, an increase in adiponectin levels, and enhanced insulin receptor sensitivity. IBM-BMT+TT enhanced HO-1 expression and increased AKT and AMPK expression in the pancreas (Li et al., 2010) and, moreover, upregulated HO-1, peNOS and pAKT levels in the kidney of these mice (Li et al., 2012). In T2DM patients, intrapancreatic autologous stem cell infusion combined with hyperbaric oxygen treatment may reduce insulin requirements (Estrada et al., 2008) and the need for oral hypoglycemic drugs (Wang et al., 2011b). In one report, autologous BMT was shown to decrease insulin requirements, which correlates with stimulation of C-peptide in T2DM patients (Bhansali et al., 2013). The report suggested that the implantation of autologous BM mononuclear cells for the treatment of T2DM is safe and effective because no side effects were noted after transplantation. This therapy can partially restore the function of islet beta-cells and maintain blood glucose homeostasis over the longer term (Hu et al., 2012).

## IBM-BMT prevents and treats osteoporosis in SAMP6 mice

SAMP6 is a kind of substrain of SAM that spontaneously develops osteoporosis early in life and is, therefore, a useful model for examining the mechanisms underlying osteoporosis (Chen et al., 2009). IL-6, TNFα and TGFβ might be involved in osteoporosis through the regulation of osteoblastogenesis and osteoclastogenesis. TNFα can stimulate the production of IL-6 by osteoblasts (originally derived from stroma cells), resulting in an augmentation of TNF-related activation-induced cytokine receptor/receptor activator of nuclear factor-kb ligand (TRAICR/RANKL), which induces osteoclastogenesis, whereas TGFα, which is produced by osteoclasts, controls the osteoblastogenesis of stroma (Mundy et al., 1995; Roggia et al., 2001). One report has indicated that strontium ranelate acts on lineage allocation of MSCs by antagonizing the age-related switch in osteoblasts to adipocyte differentiation via mechanisms involving the nuclear factor of activated T-cell (NFAT)c/Maf and Wnt signaling, resulting in increased bone formation and an attenuation of the bone loss in senescent osteopenic mice (Saidak et al., 2012). The BM microenvironment was normalized after IBM-BMT. Increased production of IL-11, IL-6, and Rank L ameliorated the imbalance between bone absorption and formation, resulting in the prevention of osteoporosis in SAMP6 (Takada et al., 2006; Ueda et al., 2007). It is accepted that RANKL, RANK, and osteoprotegerin are essential for controlling the oesteoclast development and functions in bone remodeling, and inhibition of RANKL activity by osteoprotegerin injection results in significantly reduced bone loss in arthritis (Kong et al., 1999) and osteoporosis (Mizuno et al., 1998).

## IBM-BMT ameliorates loss of cognitive ability and expression of Sirt1 on TECs in AD model mice

SAMP 8 (Butterfield and Poon, 2005) and 10 are two substrains of SAM that have been extensively used in studies as AD model mice. These mice show age-related deficits in learning and memory with/without forebrain atrophy and impaired immune response. HO-1 is a very sensitive marker of oxidative stress, and chronic over-expression of HO-1 in the AD brain, possibly in response to excessive amyloid provocation, may account for the (transferrin receptor-independent) iron overload and mitochondrial insufficiency observed in this disorder (Schipper, 2000). The higher oxidative stress status is observed to be partly caused by mitochondrial dysfunction in the SAM, resulting in the excessive production of reactive oxygen species and neurodegeneration (Chiba et al., 2009). The SAMP8 is an acceptable rodent model for cognitive deficits observed with aging, such as AD, and is found to have age-related deficits in learning and memory that could not be explained in terms of differences in sensorimotor or motivational capabilities (Flood and Morley, 1992). Moreover, beta-amyloid has been shown to play a central role in the pathophysiology of AD through the induction of oxidative stress. One report has demonstrated that antisense oligonucleotide directed against PS-1 in old SAMP8 mice improved learning and memory deficits and reduced beta-amyloid-mediated oxidative stress (Fiorini et al., 2013). Another report indicated that hydrocotyle sibthorpioides administration prevented spatial learning and memory decline by the scavenging of free radicals, up-regulating the activity of antioxidant enzymes, decreasing the level of beta-amyloid, and ameliorating dysfunction in synaptic plasticity in SAMP8 mice (Lin et al., 2013).

BM cells can increase the number of activated microglias that play a central role as APCs and reduce the amyloid deposit via phagocytosis of beta-amyloid and thereby prevent the progression of AD (Simard et al., 2006). Our report suggested that IBM-BMT normalized levels of HO-1, IL-6, IL-1β and iNOS, and ameliorated the impaired cognitive ability of SAMP 8 mice (Li et al., 2009). Initial clinical trials have suggested that non-steroidal anti-inflammatory drugs could prevent the development of ADs by inhibiting the immune response (in t' Veld et al., 2001).

SAMP10 show age-related behavioral deterioration such as deficits in learning and memory and emotional disorders (Takeda et al., 1997). These mice also show age-related changes in the brain such as brain atrophy, shrinkage and loss of cortical neurons, retraction of cortical neuronal dendrites, loss of dendritic spines, loss of synapses, impaired learning and memory, depressive behavior, accumulation of neuronal DNA damage, neuronal ubiquitinated inclusions, reduced hippocampal cholinergic receptors, decreased neurotrophic factors, decreased hippocampal zinc and zinc transporters, increased sphyngomyelinase, and elevated oxidative-nitrative stress (Shimada and Hasegawa-Ishii, 2011). The decline in learning and memory abilities of SAMP10 has been reported to be caused by a decrease in catecholamine synthesis in the cerebral cortex with aging (Shimada et al., 1993; Miyajima et al., 2013). Green tea-catechin intake prevented the experimental tumor metastasis in aged SAMP10 mice by inhibiting age-related decline in immune surveillance (Shimizu et al., 2010). One report has suggested that theanine improves the cognitive dysfunction and behavioral depression resulting from psychosocial stress (Unno et al., 2011).

We found that the percentage of CD4/TNFα T cells in the spleen of 24-week-old (but not 6-week-old) SAMP10 was significantly reduced. The thymus was significantly lighter and the percentage of CD4^+^CD8^+^ was lower in the 24-week-old SAMP10 than 6-week-old SAMP10. Real time PCR analysis showed that the expression of KGF, Aire and Sirt1 was decreased on the TECs of 24-week-old SAMP10. However, treatment with IBM-BMT improved these age-related disorders. Another report examined the finding that IBM-BMT facilitates the entry of transplanted BM-derived cells into the brain parenchyma. IBM-BMT may thus prove beneficial in the experimental treatment of psychiatric and neurological diseases (Hasegawa-Ishii et al., 2013).

## Conclusion and future direction

**Figure 2:**
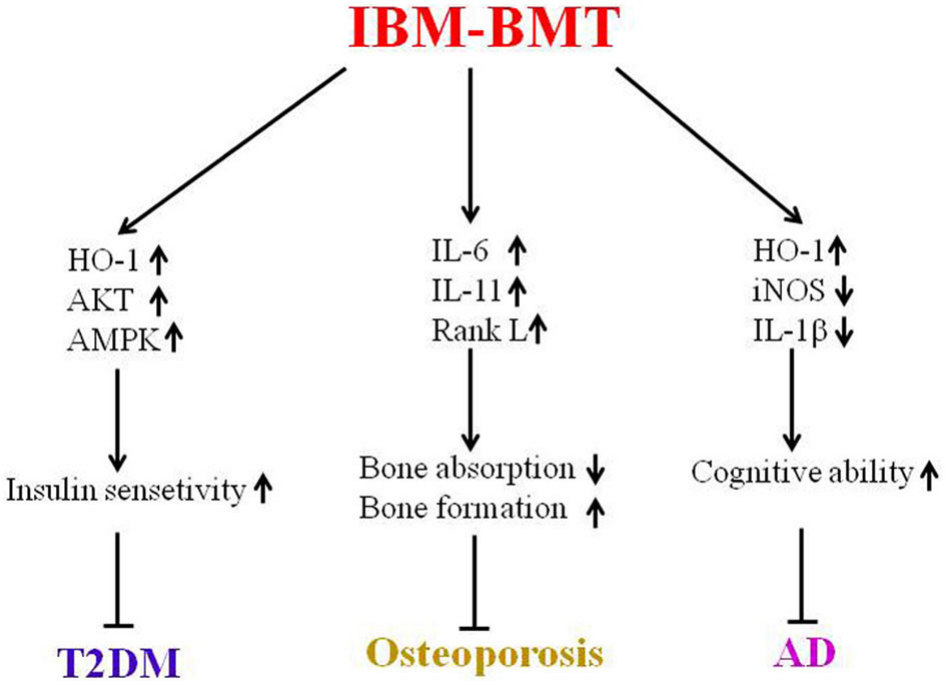
This is a summary of this review. IBM-BMT increased insulin sensitivity by upregulating the expression of HO-1, AKT and AMPK, and improving T2DM. IBM-BMT also regulated the expression of inflammatory cytokines, controlled the balance between bone absorption and bone formation, and improved osteoporosis. Furthermore, IBM-BMT regulated the expression of HO-1, iNOS and IL-1β, resulting in improved cognitive ability.

Immune dysfunction, including defective T cells and B cells may accelerate the aging process in the diabetic mice and SAMP mice. IBM-BMT has been shown to be a valuable strategy for the treatment of aging-related disorders in experimental models of T2DM, osteoporosis and AD (Figure 2). Future studies will focus on related mechanisms through which IBM-BMT improves aging-related diseases. Additionally, we will attempt to determine whether IBM-BMT can prevent or treat other aging-related diseases such as Parkinson's disease and atherosclerosis.

### Conflict of interest statement

The authors declare that the research was conducted in the absence of any commercial or financial relationships that could be construed as a potential conflict of interest.

## Abbreviations

AD: Alzheimer's disease
BM: bone marrow
BMT: bone marrow transplantation
ESC: embryonic stem cell
IBM-BMT: intra bone marrow-BMT
iPSCs: pluripotent stem cells
NFAT: nuclear factor of activated T-cell
SAM: senescence-accelerated mouse
SAMP: SAM-prone
SAMR: SAM-resistant
TECs: thymic epithelial cells
TRAICR/RANKL: TNF-related activation-induced cytokine receptor/receptor activator of nuclear factor-kb ligand
TSCs: thymic stroma cells
STZ: streptozocin
T2DM: Type 2 diabetes mellitus
TT: thymus transplantation.
